# Preventive and Proactive Coping with Bad Weather in Outdoor Sports: A Measurement Proposal

**DOI:** 10.3390/bs10040080

**Published:** 2020-04-24

**Authors:** Piotr Próchniak, Agnieszka Próchniak

**Affiliations:** 1 Department of Psychology, University of Szczecin, 71-017 Szczecin, Poland; 2 Department of Sociology, Pomeranian University, 76-200 Słupsk, Poland; agnieszka.prochniak@apsl.edu.pl

**Keywords:** coping, outdoor sports, weather

## Abstract

This article presents the Proactive and Preventive Coping with Bad Weather in Outdoor Sports Scale, a tool for diagnosing future oriented coping with bad weather in outdoor sports. A study of the psychometric properties of the Proactive and Preventive Coping with Bad Weather in Outdoor Sports Scale was conducted, with an exploratory and a confirmatory factor analysis being carried out. The first set of data (N = 326) was analysed by exploratory factor analysis, and the second set of data (N = 183) was analysed by confirmatory factor analysis. The results of factor analyses verified the two-factor structure. The Proactive and Preventive Coping with Bad Weather in Outdoor Sports Scale showed satisfactory internal consistency. The coefficient alpha reliabilities were 0.81 for the Preventive scale, and 0.80 for the Proactive scale. The divergent and convergent validity of the Preventive and Proactive Coping in Outdoor Sports Scale was indicated by correlations with scales of coping, general self-efficacy, sensation seeking and the personality NEO-FFI. The results indicate that the Proactive and Preventive Coping with Bad Weather in Outdoor Sports Scale is a valid and reliable instrument.

## 1. Introduction

Outdoor sports are the most popular types of activities undertaken in free time throughout the world. Outdoor sports activities include cycling, swimming, diving, sailing, trekking and climbing, amongst others. The main goal of outdoor sports is to improve physical, psychological and social well-being. Outdoor sports are advocated as one component of a healthy lifestyle that leads to greater longevity [1,2,3,4].

### 1.1. Effects of Weather on Outdoor Sports

Traditionally, outdoor sports are perceived as being comfortable, both physically and mentally, for the participants. One of the most significant variables determining the feeling of comfort in outdoor sports is the weather conditions. Sunny days without wind, 25% cloud cover and temperatures between 27 and 32 °C are treated as very good atmospheric conditions for recreational sport [5]. These comfort levels are in reference to the northern hemisphere. In many equatorial regions, 32 °C is the annual average temperature, so “ideal” conditions might be considered relatively different there compared to other places.

Given the importance of atmospheric conditions as a factor in outdoor sports, the body of literature that explores preferences for or expectations of outdoor sports has steadily grown. Some studies have employed a so-called climate index model, which estimates seasonality patterns on a macro-scale including different weather conditions [6,7]. Other studies analyse the environmental context for sport recreation (sea, mountains, lakes, forests), which includes weather, in personal decision-making [8,9].

Unfortunately, perfect weather does not always occur in outdoor sports. Sometimes, people practise outdoor sports in strong winds, very low or very high temperatures, fog, snow or heavy rain [10,11]. These bad weather conditions are an essential aspect of outdoor activity. Adverse weather exerts a level of influence on engagement in outdoor sport, the timing of outdoor sport, actual or planned outdoor sport and sport satisfaction [5,8,12,13].

At the physiological level, outdoor sports in bad weather can be analysed by means of processes affecting body temperature, blood pressure, changes in the hormonal system or potential consequences for physical health (e.g., hypothermia in cool-windy environments, hyperthermia in hot temperature or headache triggered by barometric changes) [14]. At the affective–cognitive level, participation in sport during adverse weather can be analysed by means of processes affecting deterioration of anticipation, decision making, perception, learning or memory. An important part of the research in this field concerns the effect of bad weather on mood disorders [14,15,16,17,18].

### 1.2. A Brief Explanation of Outdoor Sports in Risky Environments

Bad weather can have an influence on motives and satisfaction in outdoor sports. Some people do not take part in outdoor sports due to bad weather conditions. Others arrange outdoor sports which entail enduring risky weather. Surprisingly, the theories attempting to explain the engagement in unsafe outdoor activities and sports in close contact with nature are rare in regard to bad weather conditions. The most widely known research on adventure sports is Marvin Zuckerman’s work on the personality trait called sensation seeking [19]. Sensation seeking is defined as the need for “varied, novel, complex, and intense sensations and experiences, and the willingness to take physical, social, legal and financial risks for the sake of such experiences” [19] (p. 27). Much research on sensation seeking has shown scores to be associated with engagement in high-risk sports such as skydiving, hang gliding, scuba diving, surfing or climbing [20,21]. However, his theory makes no distinctions as regards which particular features of the natural environment might constitute a source for the satisfaction of the sensation-seeking need.

Reversal theory was proposed as an alternative to optimal arousal theories. In turn, Apter’s theory defines two thresholds of stimulation, the telic and the paratelic states [22]. In the telic state, individuals tend to avoid high levels of arousal, as too much arousal can make a person in this state feel anxious. In the paratelic state, the individual is playful. The paratelic state, in particular, is conducive to the undertaking of high-risk outdoor activities [23]. Like Zuckerman’s theory, though, Apter’s provides no sources on the potential hazards involved.

Csikszentmihalyi put forward a theory highlighting a balance between one’s skills and the challenge [24]. Adventure sport starts when the skills of an individual are at just the right level to manage the wilderness demands. Similar notions can be found in Priest’s theory [25].

The theory of Bandura suggests that participation in adventure sports can be determined by self-efficacy [26]. This means that self-efficacy beliefs may constitute an essential condition in the free choice of dangerous natural environments [27,28]. However, in neither case is the significance of weather conditions to the undertaking of outdoor recreational activity taken into account.

### 1.3. Coping with Bad Weather in Outdoor Sports

Research on the influence of atmospheric conditions on functioning indicate that weather phenomena such as heat waves, cold temperatures, violent winds and barometric pressure create difficult situations for the individual, exceeding the scope of his/her normal functioning [15,16,29,30,31,32]. Theories of adventure sports do explain persuasively why people seek risk in outdoor sports, but do not explain how athletes are able to cope with the stress or injury associated with harsh weather. This raises the intriguing issue of how athletes cope in such situations [33,34].

#### 1.3.1. Coping Strategies in Sport

Coping has a long tradition in the source literature. It is defined as the specific efforts, both behavioural and psychological, which people make in order to reduce or minimise stressful events [33].

Lazarus and Folkman distinguished two basic types of coping strategies, namely problem-solving strategies and emotion-focused strategies. The former concentrate on endeavours to do something active to eliminate the stressful circumstances. The latter involve attempts to regulate emotional consequences. The predominance of one strategy over the other is determined both by individual traits and the nature of the event in question [33]. The next distinction in coping strategies identified in the literature is between active and avoidance coping. Active coping strategies involve trying to change the nature of the stressor itself, while avoidance coping strategies are mechanisms characterised by effort to evade having to deal with it [35,36].

Stress coping has become one of the significant issues taken on by researchers dealing with sport. Empirical studies include the types of coping strategies used by athletes, age-related and gender differences, psychological determinants of coping in sport or coping effectiveness [37].

Researchers also proposed different tools for measuring coping in sport. For example, Besharat constructed the Sport Stress Coping Styles Scale (SSCSS) [38]. The SSCSS diagnoses the basic styles of coping in sport: approach and avoidant. Another tool, proposed by Madden and his co-workers, the Ways of Coping in Sport Scale, consists of five factors: seeking of social support; denial/avoidance; wishful thinking; effort/resolve; and emotional control [39,40]. The Athletic Coping Skills Inventory-28 (ACSI-28) measures coping skills among athletes. It is composed of seven subscales: coping with adversity; goal setting/mental preparation; concentration; peaking under pressure; confidence and achievement motivation; freedom from worry; and coachability [41]. Recently, Litwic-Kaminska and Izdebski proposed the Strategies of Coping in Sport Questionnaire (SR3S). The SR3S measures the following coping strategies in sport: settling on the goal/victory; applying mental techniques; planning/focus on activity; and seeking support [42].

#### 1.3.2. Future-Oriented Coping

The ways of coping with stress mentioned thus far refer to present or past situations. Schwarzer distinguishes forms of future-oriented coping: preventive coping and proactive coping [43].

Preventive coping concerns future events; however, it is difficult to predict the probability of a given event or the time of its occurrence. Its character is intentional. In preventive coping, the future is perceived as a source of danger; thus, the aim is to minimise future stress through taking preventive measures in the present. As a result, the individual gathers resources which will make it possible to cope effectively with future threats [34].

Proactive coping is also future-oriented but is related to future challenges. This form of coping does not arise from any negative appraisals, such as threat or loss. Positive arousal and vital energy are characteristic for proactive coping. Thus, proactive coping becomes goal management instead of risk management. In this case, the individual accumulates resources which will facilitate taking advantage of future opportunities. Proactive coping is intentional and is set on distant goals. The consequence of proactive coping is an improvement of one’s functional independence, competence, life satisfaction and engagement [34,44].

Researchers have used a variety of questionnaires to measure preventive and proactive coping. Greenglass constructed the Proactive Coping Inventory. The Proactive Coping Inventory consists of seven scales: proactive coping; preventive coping; reflective coping; instrumental support seeking; strategic planning; emotional support seeking; and avoidance coping [34]. Gan, with coworkers, translated The Proactive Coping Inventory into a 16-item instrument (the Future-Oriented Coping Inventory) to measure future-oriented coping of Chinese college students [45]. The scale consists of a proactive coping subscale and a preventive coping subscale, each consisting of eight items. The Preventive Coping Resources Inventory (PCRI) is an instrument designed to measure coping resources useful for prevention. The PCRI may provide meaningful factors useful for preventive coping, such as perceived control, self-confidence and social comfort [46].

#### 1.3.3. Preventive and Proactive Coping in Outdoor Sports

Previous scales do not concern preventive and proactive coping in relation to specific areas. There are also no questionnaires which diagnose preventive and proactive coping in a sports context.

Weather phenomena in outdoor sports are characterised by significant inconsistency and unpredictability. This is why prevention of weather risks is very important. This consists in prior accumulation of resources (in a broad sense), in order to minimise or even eliminate future losses caused by atmospheric conditions in outdoor sports. Therefore, preventive coping in outdoor sports is referred to as a future coping strategy and includes efforts to prepare for weather-induced inconveniences. The strategy concentrates on activities such as preparing the appropriate clothes, analysing weather forecasts and proceeding in accordance with guidance issued by meteorological experts on television, the internet or the radio, for instance.

Proactive coping in outdoor sports is defined as a future coping strategy and includes treating bad weather conditions in outdoor recreation as a challenge, a positive attitude towards bad weather during outdoor sports, curiosity about bad weather conditions, voluntarily seeking bad weather situations to learn about one’s mental and physical barriers or developing a new skill set in harsh weather.

That is why the aim of this article is the presentation of a new instrument to diagnose preventive and proactive coping with bad weather in sport. The following paragraphs present the phases of construction of the Preventive and Proactive Coping with Bad Weather in Outdoor Sports Scale for diagnosing people’s functioning in outdoor adventure. It seems that the design and development of a reliable and valid measure of preventive and proactive coping with bad weather in outdoor sports can further advance environmental and sport psychology research and practice. Moreover, this scale may be used by teachers to measure the effects of outdoor education. Finally, this scale can also help instructors of outdoor adventure effectively deliver diagnoses when they start to work with clients.

## 2. Method

### 2.1. Preliminary Item Pool

Work on the design of the tool started with the generation of thirty questionnaire statements describing different aspects of outdoor sports in bad weather conditions (fifteen statements for preventive coping with bad weather conditions in sport and fifteen statements for proactive coping with bad weather conditions in outdoor sport). 

A list of the thirty statements was sent to four competent assessors who are specialists in sport psychology. Each expert also has personal experience in outdoor sports. They evaluated the statements in line with a five-point Likert scale: in other words, as very good, good, average, bad or very bad. This pre-selection process reduced the number of statements to twenty-two (nine statements for preventive coping in outdoor sport and thirteen statements for proactive coping in outdoor sport).

### 2.2. Participants

Data were collected from three separate groups of respondents. The first sample included 326 Polish respondents (151 women and 175 men) (M age = 23.40; SD = 2.50). The respondents in this group practised the following outdoor sports: cycling (48.00%); swimming (45.00%); kayaking (27.70%); skiing (19.00%); climbing (12.00%); scuba diving (12.50%); sailing (10.00%); snowboarding (9.00%); windsurfing (6.90%); skydiving (4.90%); paragliding (2.50%); other (19.00%). The sum of percentages is higher than 100 because some respondents practised more than one outdoor sport.

Data obtained from this sample were examined using exploratory factor analysis, reliability and item-total correlations of the new scale.

The second sample included a separate group of 183 Polish respondents (88 women and 95 men) (M age = 22.20; SD = 2.70). The respondents in this group practised the following outdoor activities: skiing (33.00%); kayaking (32.00%); snowboarding (15.50%); climbing (12.40%); scuba diving (12.00%); sailing (6.00%); skydiving (3.90%); other (17.00%). The sum of percentages is higher than 100 because some respondents practised more than one outdoor sport.

Data obtained from this sample were examined using confirmatory factor analysis.

The third sample consisted of 237 respondents: 120 women (51%) and 117 men (49%). Participants’ ages ranged from 19 to 26 (M age = 22.55; SD = 3.60). The respondents practised different forms of outdoor activities: cycling (43.00%); running (38.20%); kayaking (32.00%); horse riding (16.40%); skiing (13.20%); mountain climbing (11.70%); mushroom picking (9.80%); snowboarding (7.50%); fishing (7.40%); orienteering (7.30%); sailing (5.70%); scuba diving (3.90%); skydiving (3.20%); windsurfing (2.50%); paragliding (1.60%); and other (34.00%). Because some of participants practised more than one outdoor activity, the sum of percentages is higher than 100.

Data obtained from this sample were focused on the convergent and divergent validity of the Preventive and Proactive Coping with Bad Weather in Outdoor Sports Scale

### 2.3. Procedure

Respondents who practised outdoor sports were approached by the researchers. Those who agreed completed the questionnaires anonymously. Participation in the research was voluntary. All the participants were selected on the basis of the following criteria: (a) they operated in the province of Pomerania (Poland); (b) they had more than one year of experience in outdoor sports; (c) they were highly interested in participating in the research. The respondents were asked to complete a consent form, to carefully read the directions for the scale before responding and to raise their hands if they had any questions.

### 2.4. Measures

The list of twenty-two statements was administered to the first sample of respondents. The language of the statements was Polish. Participants completed a preliminary pool of 22 coping items. The respondents’ task was to evaluate each of the twenty-two statements and select one of the opinions from a four-level, Likert-type scale. The options provided were from (1), definitely not true, to (4), definitely true. The scale also included basic demographic information such as gender, age and type of outdoor sport. Based on the results obtained in the exploratory factor analysis (EFA), confirmatory factor analysis (CFA) was conducted on scale scores in the second group of participants.

The Polish versions of five measures related to coping with difficult situations were administered to the third group of participants in addition to the Preventive and Proactive Coping with Bad Weather in Outdoor Sports Scale.

#### 2.4.1. Proactive Coping Inventory (PCI)

The Proactive Coping Inventory is a multidimensional inventory consisting of 55 items grouped in seven subscales [47,48]. In this study, only two of the subscales were used: the 14-item Proactive Coping Scale (Cronbach’s α = 0.87) and the 10-item Preventive Coping Scale (Cronbach’s α = 0.79).

#### 2.4.2. Coping Inventory for Stressful Situations (CISS)

The CISS contains three 16-item scales which assess task-oriented coping (task scale) (Cronbach’s α = 0.90), emotion-oriented coping (emotion scale) (Cronbach’s α = 0.82), and avoidance-oriented coping (avoidance scale) (Cronbach’s α = 0.78) [35,49].

#### 2.4.3. The Generalised Self-Efficacy Scale (GSES)

The GSES is 10-item scale, included in one factor [50,51]. It measures how much respondents believe they can achieve their tasks in the face of difficulties (Cronbach’s α = 0.85).

#### 2.4.4. NEO-FFI Personality Test

The fourth questionnaire used was the NEO-FFI personality test [52]. The NEO-FFI measures the five major dimensions: neuroticism (N), extroversion (E), openness to experience (O), agreeableness (A), and conscientiousness (C). This questionnaire contains 60 questions: 12 statements for each factor. Coefficients’ alpha reliability for the NEO-FFI were: neuroticism (Cronbach’s α = 0.80), extraversion (Cronbach’s α = 0.77), openness to experience (Cronbach’s α = 0.68), agreeableness (Cronbach’s α = 0.68), and conscientiousness (Cronbach’s α = 0.82).

#### 2.4.5. Sensation Seeking Scale IV

The last instrument to be used was the Sensation Seeking Scale IV. The subscales were the same as in the original version. In this study, the following subscales of the SSSIV were used: the 14-item thrill and adventure seeking subscale (Cronbach’s α = 0.79), the 15-item experience seeking subscale, (Cronbach’s α = 0.75), the 17-item disinhibition subscale (Cronbach’s α = 0.73), and the 18-item boredom susceptibility subscale (Cronbach’s α = 0.70) [53].

## 3. Results

### 3.1. Exploratory Factor Analysis

Prior to factor extraction, the Kaiser–Meyer–Olkin (KMO) measure of sampling adequacy and Bartlett’s test of sphericity (BTS) were applied to the data. The Kaiser–Meyer–Olkin supported factorability, R = 0.85; Bartlett’s test of sphericity indicated a breach of sphericity χ^2^(231) = 2327.954; *p* < 0.01. An exploratory factor analysis using the maximum-likelihood method of parameter estimation was performed for twenty-two statements on the data from the first sample of 326 respondents and resulted in two factors.

The quality of the items that composed the two-factor solution was also analysed using the classification of Comrey and Lee [54]. Thus, as to the items’ quality, three statements for preventive coping in outdoor sport loaded below 0.53 and were rejected. Similarly, five statements for proactive coping in outdoor sport loaded below 0.53 and were rejected. Fourteen statements were accepted for the final version of the Preventive and Proactive Coping with Bad Weather in Outdoor Sports Scale. See Table 1.

Additionally, parallel analysis (PA) was carried out as an additional criterion to confirm the number of distinguishing factors [55]. See Table 2.

### 3.2. Confirmatory Factor Analysis

The factor structure identified via exploratory factor analysis was then tested with confirmatory factor analysis and validated in the sample of 183 respondents. We began with a confirmatory factor analysis (CFA) on the 14 items using maximum-likelihood estimation. Confirmatory factor analysis requires multivariate normality. Univariate skewness and univariate kurtosis values ranges from −0.360 to 0.108 and −0.425 to −0.722 to 0.525, respectively. The value of Mardia’s normalised multivariate estimate of multivariate kurtosis is 30.613. Kurtosis values greater than 3.00 in magnitude may indicate that a variable is not normally distributed [56]. Our findings indicate that the data are not multivariate normal. In order to address the issue of multivariate non-normality, the Bollen–Stein bootstrap procedure was employed [57]. Using the Bollen–Stein bootstrap with 95% bias corrected confidence levels to estimate the significance of the chi-square estimate; the model fit of the data is χ^2^/df = 132.756, *p* < 0.000; CFI = 0.924; RMSEA = 0.068.

An excellent fitting model has a small, non-significant chi-square value [58]. Our findings—a χ^2^/df = 77 value of 132.756 and a probability of 0.00 (*p* < 0.001)—thereby suggest that the fit of the data to the hypothesised model is not adequate. 

The χ^2^ statistic is very sensitive to large samples [59]. Therefore, other goodness-of-fit indices are usually used in addition to the statistic. Bentler suggests that the comparative fit index (CFI) should be the index of choice because CFI tends to properly estimate the fit in small samples (our sample is rather small) [60]. In this study, the CFI index is 0.924, which is greater than the cut-off value of 0.90 [61]. The value of the CFI index suggests that the hypothesised model represented an adequate fit to the data.

Another good indicator of model fit adequacy is the root-mean-square error of approximation RMSEA. Browne and Cudeck suggest that reasonable errors of approximation in the population can tolerate RMSEA values up to 0.08 [62]. In this study, the RMSEA = 0.068, which is less than 0.08, indicating a good fit.

### 3.3. Correlations of the Preventive and Proactive Coping with Bad Weather in Outdoor Sports

#### Scale with Other Scales

Table 3 presents the results for correlations between the coping scales, the Self-Efficacy Scale, the Sensation Seeking Scale, the NEO-FFI Scale and the Preventive and Proactive Coping with Bad Weather in Outdoor Sports Scale. Pearson’s r correlation coefficient was used. We evaluated the normality of the variables before selecting the correlation test. See Table 3.

The Preventive Coping with Bad Weather in Outdoor Sports subscale correlates positively with the following scales: all coping scales; the Self-Efficacy Scale; and the personality scales for agreeableness and conscientiousness. It correlates negatively with the Experience Seeking Scale, the Disinhibition Scale and the Boredom Susceptibility Scale (*p* < 0.05).

The Proactive Coping with Bad Weather in Outdoor Sports Sub-scale correlates positively with the coping scales (except the Emotion Oriented Coping Scale), the Self-Efficacy Scale, and the Extraversion and Thrill and Adventure Seeking Scale (*p* < 0.05).

## 4. Discussion

The Preventive and Proactive Coping with Bad Weather in Outdoor Sports Scale seems to be a valid measure of coping with bad weather in outdoor sports. The findings support the presence of independent dimensions of coping with harsh weather. Current research confirms the previous studies of Gan, which found a two-dimensional structure of preventive and proactive coping [45].

The items load on two factors with good internal consistency (preventive coping *α* = 0.81; proactive coping *α* = 0.80). The item–total correlations for preventive coping were between 0.51 to 0.65, and for proactive coping were between 0.40 to 0.72.

The Preventive and Proactive Coping with Bad Weather in Outdoor Sports Scale seems to diagnose actual practised outdoor sports rather than relying on preferences concerning the choice of sport in severe atmospheric conditions. Moreover, assessment of preventive and proactive coping in outdoor sports avoids concentration on specific sports or recreations, which can promote more precise comparisons between athletes who prefer highly varied outdoor sports.

The Preventive and Proactive Coping with Bad Weather in Outdoor Sports Scale demonstrates convergent and divergent validity in terms of its expected correlations with other measures.

The Preventive Coping and Proactive with Bad Weather in Outdoor Sports Scale correlates with coping scales. This result suggests that preventive and proactive coping in outdoor sports can be an aspect of the broader construct of coping. Preventive and proactive coping with bad weather whilst practising outdoor sports also correlates with self-efficacy. This result means that individuals with a high self-efficacy can protect themselves against the possible negative consequences of harsh weather and, simultaneously, they treat harsh weather as a source of challenge.

Personality traits also correlate to coping with harsh weather. The correlations between variables are rather poor. Thus, interpretation of correlational findings must be quite cautious. The results indicate that extraversion correlates with proactive coping with bad weather. According to Carver and Connor Smith, extraversion, as a personality trait, expresses the level of energy and activity [36]. Given that coping with harsh weather conditions requires high energy and activity levels, the result is both clear and understandable.

Preventive coping with bad weather in outdoor sports correlates with agreeableness and conscientiousness. Agreeableness means that the person is influenced by other people. In the context of coping with severe weather, the result for this trait is also understandable [36]. Athletes with a high score for agreeableness take weather forecasts seriously and try to adapt their own behaviour in accordance with the suggestions of the meteorological experts. Conscientiousness expresses the level of organisation, the undertaking of ambitious goals and perseverance in achieving them. For people scoring high for this trait, the future is more important than the present [36]. In turn, prevention also relates to events that may occur in the future. Conscientious athletes are geared not only towards the accomplishment of their purposes or projects, but also both to analysing the risks associated with their aspirations and to trying to take protective measures against these hazards, which include dangers posed by the weather.

The correlations between sensation seeking and coping with bad weather are rather low. Thus, we must be cautious in interpreting the findings. The correlation between the Thrill and Adventure Seeking Scale and proactive coping in outdoor sports seems to be understandable. TAS express a desire to engage in risky sports [19]. Severe weather presents a high-risk situation. This type of situation is thus a challenge and, as such, attractive to athletes with a high need for risk taking. However, sensation-seeking athletes do not take warnings about the dangers inherent to such activities seriously and may therefore have more weather-related accidents in practising outdoor sports.

Interestingly, other sub-scales of sensation seeking correlate negatively to preventive coping. Preventive coping with bad weather in outdoor sports negatively correlates with sub-scales of sensation seeking (except thrill and adventure sensation seeking). Low scores on sensation seeking express risk avoidance [19,63]. Harsh weather conditions are highly intensive stimuli. It is thus no surprise that a person scoring low on this trait will respond well both to a potential deterioration in weather conditions by employing preventive coping strategies when it comes to direct confrontation with high winds, heavy rain and intense cold.

## 5. Limitations and Suggestions for Future Research

The participants in this study were young rather than old people. They are a select group and not a representative sample of the population. This fact makes generalisation difficult. Future research should assess other groups of people as well. Future research should also explain the role of gender in coping with bad weather in outdoor sport.

Under this study, only some of the variables which may well be of significance to coping in severe weather conditions were subjected to analysis. Future research might encompass some of the concepts of dealing with adverse situations which were not incorporated, such as, for example, psychological capital [64], core self-evaluations [65] or positive orientation [66].

The data were collected in Poland. Several items concern weather phenomena such as fog or stormy weather, which are characteristic for Poland. It might be difficult for people from other geographical or weather regions to respond to these statements.

An important limitation of the present study is that some correlations are low and should be considered more as a trend.

Constructing scales that can assess coping with severe weather in outdoor sports is becoming important. Surprisingly, in the literature, there have been few scales that measure dealing with difficulties within natural environments. To fill this gap, a new scale was constructed: *the Preventive and Proactive Coping with Bad Weather in Outdoor Sports Scale.* It seems that this scale may help to explain voluntary practising of sport in contexts where the weather is a factor.

## Figures and Tables

**Table 1:** Factor loadings for the Preventive and Proactive Coping with Bad Weather in Outdoor Sports Scale.

No.	ITEMS:	M	SD	Preventive Coping	Proactive Coping	I-T
1	I continue outdoor sport even when it is raining or cold because I want to check my breaking point	2.53	0.89		0.53	0.40
2	I check the weather forecast before undertaking outdoor sport	2.29	0.88	0.78		0.65
3	I treat confrontation with bad weather as a battle	2.45	0.87		0.80	0.69
4	I protect myself against possible weather changes when I intend to set off on a physical activity in a natural environment	2.44	0.80	0.69		0.53
5	I like struggling with the wind during outdoor sport	2.60	0.93		0.69	0.56
6	Running on the beach in stormy weather evokes positive excitement in me	2.79	0.97		0.57	0.44
7	I take very seriously weather forecasts about upcoming weather conditions during sport in nature	2.38	0.77	0.78		0.64
8	Cool air is not a problem for me when taking part in outdoor sport	2.72	0.83		0.62	0.48
9	I check the weather in places where I intend to take part in outdoor sport	2.57	0.95	0.70		0.56
10	Outdoor sport in the fog increases my curiosity	2.37	0.89		0.62	0.49
11	I like to prepare myself before doing sports in nature, so as not to be surprised by the weather conditions	2.77	0.81	0.66		0.51
12	Heavy rain during outdoor sports inspires my enthusiasm to overcome difficulties	2.28	0.86		0.82	0.72
13	During outdoor sport, I follow the advice of weather services when they suggest caution in connection with the worsening weather	2.32	0.85	0.66		0.52
14	Rain or snow does not bother me when I care about experiencing adventure while doing outdoor sports	2.57	0.91		0.60	0.47
	Variance (%)			22.10	26.10	
	Cronbach’s *α* =				0.80	
	Cronbach’s *α* =			0.81		

**Table 2:** Parallel analysis for the Preventive and Proactive Coping with Bad Weather in Outdoor Sports Scale.

Variable	Sample N = 326	
	PCA	PA
1	3.56	1.46
2	3.18	1.35
3	0.98	1.26
4	0.91	1.19
5	0.88	1.12
6	0.76	1.06
7	0.67	1.01
8	0.59	0.95
9	0.49	0.89
10	0.45	0.84
11	0.42	0.79
12	0.38	0.73
13	0.35	0.67
14	0.29	0.60

PCA—Principal Component Analysis; PA—Parallel Analysis; The parallel analysis showed a two-factor solution.

**Table 3:** Correlations between the coping scales, the Self-Efficacy Scale, the NEO-FFI Scale, the Sensation Seeking Scale, and the Preventive and Proactive Coping with Bad Weather in Outdoor Sports Scale.

Scales	Preventive Coping with Bad Weather	Proactive Coping with Bad Weather
Preventive Coping	0.47 *	0.30 *
Proactive Coping	0.25 *	0.45 *
Task Oriented Coping	0.37 *	0.25 *
Emotion Oriented Coping	0.25 *	0.03
Avoidance Oriented Coping	0.19 *	0.22 *
Self-Efficacy	0.19 *	0.28*
Extraversion	0.09	0.19 *
Neuroticism	0.02	−0.03
Agreeableness	0.26 *	0.00
Conscientiousness	0.29 *	0.11
Openness to Experience	0.01	0.14
Thrill and Adventure Seeking	−0.01	0.25 *
Experience Seeking	−0.27 *	0.10
Disinhibition	−0.18 *	0.04
Boredom Susceptibility	−0.15 *	−0.05

* *p* < 0.05.
